# Strong Amphoteric Adsorption of Reactive Red-141 onto Modified Orange Peel Derivatives: Optimization, Characterization, and Mechanism

**DOI:** 10.3390/polym17131875

**Published:** 2025-07-04

**Authors:** Behlul Koc-Bilican, Ismail Bilican, Hakan Çelebi

**Affiliations:** 1 Department of Molecular Biology and Genetics, Faculty of Science and Letters, Aksaray University, 68100 Aksaray, Türkiye; behlulkoc@aksaray.edu.tr; 2 Department of Electronics and Automation, Technical Vocational School, Aksaray University, 68100 Aksaray, Türkiye; ismailbilican@aksaray.edu.tr; 3 Department of Environmental Engineering, Faculty of Engineering, Aksaray University, 68100 Aksaray, Türkiye

**Keywords:** orange peel derivatives, reactive red-141, sustainable adsorbents

## Abstract

This study investigates the adsorption performance of Reactive Red-141 (ReR-141) using three modified orange peel derivatives: raw orange peel (ROP), oil-free orange peel (NOOP), and cellulose extract (CE). The adsorbents were prepared through sequential treatments and characterized by scanning electron microscopy, energy-dispersive X-ray spectroscopy, and Fourier-transform infrared spectroscopy to investigate their surface morphology and functional groups. Batch adsorption experiments were conducted under varying conditions of pH, temperature, time, and adsorbent amount. NOOP displayed the highest adsorption capacity (99.72% removal efficiency), followed by CE (86.99%) and ROP (77.55%), under optimal conditions. The adsorption kinetics followed a PSO model, while the equilibrium data were best described by Langmuir, indicating monolayer adsorption. Thermodynamic factors confirmed that the process was self-generated and primarily determined by physisorption. Desorption studies using 0.2 M NaOH demonstrated that NOOP retained 98.16% efficiency after three cycles, indicating its strong reusability. The adsorption mechanism is determined by different interactions, such as electrostatic forces, H-bonding, and π–π stacking. These findings suggest that orange peel derivatives, particularly NOOP, serve as optimal and environmentally sustainable adsorbents for the yield of ReR-141 from synthetic aqueous media.

## 1. Introduction

Climate changes, the COVID-19 pandemic, and human activities are profoundly affecting the global ecosystem. Increasing population and industrialization exacerbate water pollution, making the provision of clean water supplies more challenging [1,2]. This situation has led scientists to investigate water pollution and develop preventive methods. Anthropogenic activities lead to the release of different kinds of chemical substances and waste. Industrial waste typically contains toxic pollutants such as heavy metals, pesticides, dyestuff, inorganic/organic gases, volatile compounds, and chemical fertilizers [3,4]. Dyestuffs constitute a significant group of pollutants in water pollution and are broadly used in the cosmetics, food, paper, and textile industries. Currently, more than 100,000 types of dyestuffs exist, and it has been determined that 54% of dyestuffs in aquatic environments originate from the textile industry [5,6,7,8]. The textile sector accounts for 20% of global water pollution, second only to the petroleum industry [9].

Each year, 800,000 tons of dyestuffs are produced, with reactive azo dyestuffs comprising 70% of the total. Of these, 10–15% are lost in the environment, causing significant damage to ecosystems [10]. Reactive Red-141 (ReR-141) belongs to the reactive azo dyestuff group due to the presence of sulfonic (SO_3_^2−^) and carbonaceous (–COO−) species in its structure. Due to its high solubility, reactivity, and toxicity, ReR-141 poses a significant environmental threat and serious risks to human health and ecosystems [8]. Therefore, researchers have been developing functional methods for the yield of dyestuffs like ReR-141 for many years [6,11]. Various techniques, including adsorption, biological degradation, advanced oxidation, photocatalysis, ozonation, and membrane filtration, have been applied [12]. However, factors such as high costs, complex operational processes, and secondary pollution limit the efficiency of these methods [13]. Adsorption is a favored technique since it is inexpensive, simple to use, and reusable. The properties of the adsorbent material have a significant impact on its ability to adsorb. Recently, natural waste products have been studied as potential adsorbents [14,15]. Citrus fruits are widely consumed worldwide, with oranges being the most significant representative (~61%) [16]. Orange peels constitute 50–60% of the fruit’s weight and contain components such as cellulose and pectin, making them a promising adsorbent material [17].

Today, global nations face two major challenges: excessive resource consumption and the growing volume of waste—both of which hinder sustainable water and sanitation management. The United Nations addresses these concerns through Sustainable Development Goals (SDG) 6 and 12. This study aligns with SDG 12 by evaluating the sustainable use of orange peel waste, while its relevance to SDG 6 lies in the investigation of ReR-141 dye removal through adsorption. Raw orange peel, like many unprocessed natural adsorbents, generally exhibits limited pollutant removal capacity. However, numerous natural materials—often classified as waste—have been effectively employed for dye removal from wastewater. In our previous studies, we also explored the adsorption potential of such discarded materials in their unmodified forms. In this study, orange peel, a significant component of fruit waste, is evaluated within the framework of a “waste-to-waste removal” approach. Especially during the rise of the zero-waste movement, it has been emphasized that seemingly worthless materials can be repurposed. From the perspective of industrial symbiosis, such organic wastes may also find applications in fields such as medicine, cosmetics, and water treatment rather than being disposed of. According to the literature, surfaces with a water contact angle ≥ 150° and low contact angle hysteresis are termed “super adsorbents” due to their rough morphology and high adsorption performance. In contrast, orange peel has a water contact angle of 0° [18], which limits its intrinsic adsorption capacity. Nevertheless, despite low equilibrium adsorption values (q_e_), high removal efficiencies have been reported—likely attributed to the peel’s abundance of functional groups and bioactive compounds, including naringenin, tangeretin, hesperidin, sinensetin, cellulose, hemicellulose, pectin, lignin, and various phenolics. These oxygen-containing groups enhance the interaction between the adsorbent and pollutants. Additionally, the bio-organic structure of orange peel enables adaptable adsorption performance under different conditions, including pH, contact time, and temperature. While surface modification processes may enhance adsorption by improving porosity, hydrophilicity/hydrophobicity, and thermal/microbial resistance, they may also damage the structural integrity of the material—causing pore deformation and inconsistent efficiency. To mitigate such drawbacks and reduce treatment costs, chemical usage, and secondary pollution, this study emphasizes the use of minimally processed materials. Accordingly, orange peel was prepared and tested in three different forms: raw orange peel (OP); OP with bioactive oils and compounds removed to improve adsorption without altering surface morphology; and synthesized cellulose extracted from OP, based on its known adsorptive potential. All three materials were evaluated for their ReR-141 dye-removal capabilities under identical batch adsorption conditions using laboratory-scale synthetic solutions. These tests were conducted without subjecting the materials to intense chemical or thermal treatment, thereby preserving their natural surface integrity. The core aim of this study is to develop a low-cost, eco-friendly adsorption strategy using waste-derived materials—minimizing both financial and environmental impacts. In doing so, this work supports the sustainable transformation of agricultural waste into functional resources for wastewater treatment applications.

Building on this foundation, this study focuses on a comprehensive evaluation of the adsorption potential of the three prepared materials. This research involves the following: (i) characterization of raw OP, bioactive compound-removed OP, and synthesized cellulose using scanning electron microscopy (SEM), energy-dispersive X-ray spectroscopy (EDX), and Fourier-transform infrared spectroscopy (FTIR); (ii) assessment of ReR-141 removal efficiency under varying conditions, including pH, adsorbent dosage, temperature, and contact time; (iii) comparison of the desorption behavior of the three adsorbents under optimal conditions to evaluate reusability; (iv) application of kinetic and isotherm models to describe the adsorption process; and (v) investigation of the potential adsorption mechanisms involved. This multi-faceted approach aims to provide deeper insights into the effective use of unmodified or minimally processed waste-derived materials for dye removal. Moreover, by integrating orange peel into the waste valorization cycle, this study highlights its transformation into a high-value product—contributing to circular and sustainable waste management strategies. Our findings are expected to serve as a valuable scientific reference for future research in environmental remediation and green material development.

## 2. Materials and Methods

### 2.1. Chemical Usage in the Adsorption Process

The following materials were acquired from Sigma-Aldrich (St. Louis, MO, USA) and utilized in the experiments: methanol, chloroform, ReR-141, petroleum ether (75% analytical purity), HCl (37% analytical purity), NaOH (98% analytical purity), and H_2_SO_4_ (95% analytical purity). The pH adjustments of the solutions were performed using 0.1 M H_2_SO_4_ (95%) and NaOH (98%). A Millipore Elix Advantage 5-Synergy UV device (Merck KGaA, Darmstadt, Germany) was used to provide ultrapure water for the preparation of all solutions. For working solutions with a ReR-141 concentration of 10 mg/L, a 1000 mg/L stock solution was used. The general properties and chemical structure of ReR-141 are presented in Table S1. A Shimadzu UV-1280 brand spectrophotometer (Shimadzu Corporation, Türkiye Branch Office, İstanbul, Türkiye) was used to quantify the concentration of ReR-141 at an absorption wavelength (λmax) of 544 nm.

### 2.2. Preparation of ROP, NOOP, and CE

Raw orange peels (ROPs), used as an adsorbent, were obtained from households, cafés, and marketplaces in Aksaray, Turkey. The foreign materials on the ROP surface were removed by washing with pure water. The washed ROPs were dried in a Memmert oven at 50 °C for 48 h to remove moisture. The dried ROPs were ground using an Arnica GH21520 grinder (Arnica Marketing Inc., Istanbul, Türkiye) and then sieved (mesh No. 10) to obtain a powder with a maximum particle size of 2 mm.

To remove oil content, 100 g of powdered ROP was treated with petroleum ether for 24 h at room temperature, using a Soxhlet extractor with intermittent stirring. The suspension was then filtered through a 110 μm pore-size filter membrane. The petroleum ether in the filtrate was evaporated under vacuum at 50 °C. All obtained isolates were stored at +4 °C until used in experiments. The residue retained on the filter membrane, free of oil (NOOP), was dried at 50 °C for 24 h to ensure complete removal of residual ether.

The powdered ROPs were treated with a 2 M HCl solution at 100 °C for 3 h to remove minerals and other residues from their structure. After acidic treatment, the samples were washed with distilled water until a neutral pH was achieved. The samples were then dried at 50 °C for 24 h in an oven. To remove proteins and non-cellulosic materials, the dried samples were further treated with a 2 M NaOH solution at 120 °C for 8 h. After alkaline treatment, the samples were filtered and then washed with distilled water, and neutral pH was ensured. Finally, to eliminate remaining pigments and waxes, the samples were treated with a methanol–water–chloroform mixture (1:2:1 volume). This process improved the purity of cellulose. The obtained cellulose (CE) fibers were dried in an oven at 50 °C for 24 h.

### 2.3. Characterization of ROP, NOOP, and CE

The microscopic surface and elemental distribution analyses of ROP, NOOP, and CE were conducted using a SEM (Fei Quanta FEG 250, 10 kV, Ciqtek, Beijing, China) combined with EDX (Octane Pro, EDAX, AMETEK, Inc., Mahwah, NJ, USA). To identify the functional groups present on the surfaces of ROP, NOOP, and CE before and after ReR-141 adsorption, FTIR spectroscopy (Perkin Elmer Spectrum FTIR C96108, PerkinElmer Health and Environmental Sciences Ltd., İstanbul, Türkiye) was used in the spectral range of 4000 to 500 cm^−1^.

### 2.4. Adsorption and Desorption Experiments

Adsorption studies were conducted in batch mode to investigate the removal performance of ReR-141 dyestuff. In the first stage of this study, a calibration curve was created for ReR-141 in the amount range of 0.05–10 mg/L. Throughout this study, the inlet and outlet dyestuff concentrations were measured at 544 nm, the maximum absorbance wavelength for ReR-141, using a Shimadzu UV-1280 spectrophotometer. To evaluate the adsorption performance of ROP, NOOP, and CE, key process factors (pH, adsorbent amount, temperature, and time) were investigated. The adsorption experiments were carried out under constant conditions of 20 ± 2 °C, an experimental volume of 50 mL, and a fixed ReR-141 concentration of 10 ± 3 mg/L. Homogeneous mixing was ensured using a ZHICHENG (Shanghai, China) platform shaker at a constant stirring speed of 150 ± 5 rpm.

(1) $$q_{e}=\frac{\left(C_{0}-C_{e}\right)\times V}{1000\times m}$$

(2) $$ReR-141\;Removal\left(\%\right)=\frac{C_{0}-C_{e}}{C_{0}}\times 100$$

Adsorption studies were conducted in batch mode to investigate the removal performance of ReR-141 dyestuff. In the first stage of this study, a calibration curve was created for ReR-141 in the amount range of 0.05–10 mg/L. Throughout this study, the inlet and out-let dyestuff concentrations were measured at 544 nm, the maximum absorbance wave-length for ReR-141, using a Shimadzu UV-1280 spectrophotometer. To evaluate the ad-sorption performance of ROP, NOOP, and CE, key process factors (pH, adsorbent amount, temperature, and time) were investigated. The adsorption experiments were carried out under constant conditions of 20 ± 2 °C, an experimental volume of 50 mL, and a fixed ReR-141 concentration of 10 ± 3 mg/L. Homogeneous mixing was ensured using a ZHICHENG platform shaker at a constant stirring speed of 150 ± 5 rpm.

To determine the equilibrium adsorption process and the optimal removal efficiency of ReR-141, batch tests were conducted under varying conditions, including ROP, NOOP, and CE dosages (0.1–5 g); pH (2–12); contact time (5–150 min); and temperature (20–40 °C). Throughout all experiments, pH monitoring and adjustments were performed using a HACH HQ440D multiparameter meter. For pH adjustments, acid and base agents were used. The batch experiments were conducted in triplicate, ensuring a 95% confidence level (standard deviation ≤ 5%). The adsorption capacity (q_e_) (mg/g) and removal efficiency (%) were determined using Equations (1) and (2), where C_0_ and C_e_ (mg/L) represent the initial and final ReR-141 concentrations, respectively; V (L) is the solution volume; and m (g) is the amount of ROP, NOOP, or CE.

To understand the adsorption behavior and interaction mechanism of ROP, NOOP, and CE with ReR-141, various mathematical models were evaluated based on the obtained experimental data. Mathematical models were developed using four distinct kinetic and isotherm models. Additionally, for the adsorption process conducted under different temperature conditions, thermodynamic parameters were calculated. All equations used in the kinetic, isotherm, and thermodynamic (Arrhenius and Van’t Hoff equations) evaluations are summarized in Table S2.

Based on the optimum adsorption data, the reusability experiments of ROP, NOOP, and CE were conducted under acidic (pH:4) and basic (pH:12) conditions, using 0.2 M HCl and NaOH eluents. The optimized three adsorbents were first washed with pure water. The dyestuff-loaded ROP, NOOP, and CE were then placed in Erlenmeyer flasks containing 50 mL of eluent and ultrasonically tested at a constant temperature of 35 °C for 60 min. Following this procedure, pure water was used for a second filtering and washing of ROP, NOOP, and CE. Finally, they were dried at 60 °C for 2 h. Up to five cycles of adsorption studies were conducted using the acquired ROP, NOOP, and CE. For each case, the desorption percentage was calculated using Equation (3), where Cd (mg/L) represents the concentration of ReR-141 dyestuff in the desorbed medium.

(3) $$Desorption\;efficiency\left(\%\right)=\frac{C_{d}}{C_{0}-C_{e}}\times 100$$

## 3. Results

### 3.1. Functional Group on the Surface of ROP, NOOP, and CE

**Figure 1:**
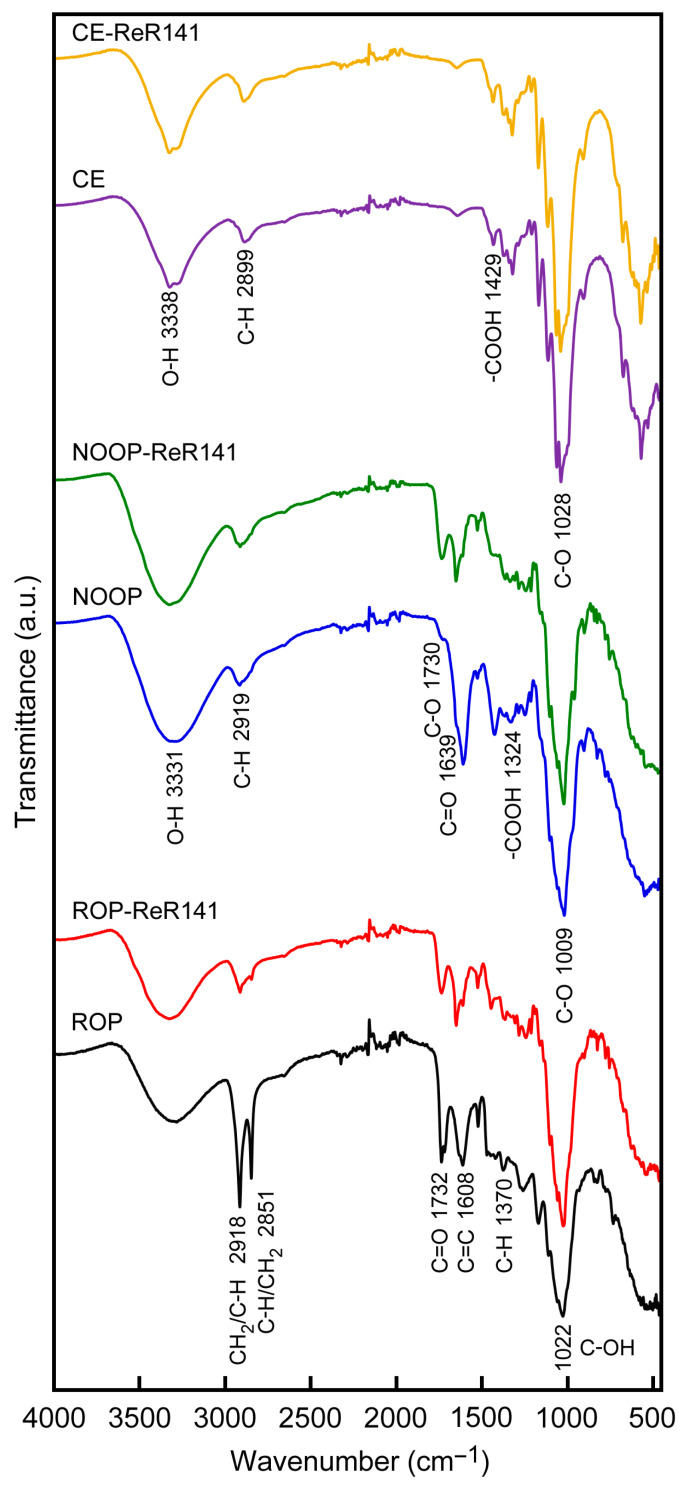
FTIR spectra of ROP, NOOP, and CE before and after the adsorption of ReR141.

As a result of the FTIR analysis applied to three different forms of orange peel, the functional groups responsible for adsorption, such as hydroxyl, carbonyl, carboxylic, and amine groups, were identified in each form. In the FTIR spectra of ROP, NOOP, and CE, shown in Figure 1, several main peaks were observed, reflecting the complex structure of raw orange peel. O–H, C–H, C=C, C–O, C=O, and –COOH are the main functional groups of ROP, NOOP, and CE, where O–H stretching represents lignin and cellulose, while C–H stretching may originate from aliphatic groups in the natural structure of ROP. The peak observed at 3324 cm^−1^ for ROP can be attributed to hydrogen bonds in pyranose rings (sugar-based components) [19]. The peaks at 2918 cm^−1^ and 2851 cm^−1^ can be assigned to aliphatic C–H and CH_2_ vibrations corresponding to hemicellulose and cellulose [20]. The peak at 1732 cm^−1^ corresponds to the COO and C=O vibrations of cellulose, pectin, and lignin in ROP [21]. The peak at 1608 cm^−1^ indicates C=C stretching in aromatic components of ROP. The band at 1606 cm^−1^ is ascribed to aliphatic and unsaturated aromatic compounds, whereas the band at 1737 cm^−1^ is ascribed to carbonyl groups in ester bonds. The fundamental structure of lignocellulosic ROP is formed by C–H bending, which is represented by peaks in the region of 1370 cm^−1^ to 1253 cm^−1^. On the other hand, the peak at 1022 cm^−1^ can suggest the presence of the C–OH group [18,22]. For NOOP, as shown in Figure 1, specific stretches occurred at different peak positions. O–H at 3331 cm^−1^, C–H at 2919 cm^−1^, C=C at 1639 cm^−1^, C–O at 1730 cm^−1^ and 1050 cm^−1^, and –COOH at 1423 cm^−1^ and 1324 cm^−1^ were determined [23]. In the obtained peak distributions, all the main functional groups of NOOP, which had undergone the removal of volatile and essential oils, were recorded with sharper peaks than those in the ROP spectrum. This supports the higher adsorption capacity of NOOP for ReR-141 compared to ROP. Additionally, the fundamental principle of chemical modification is to remove unwanted components and increase functional groups that can synchronize with the dyestuff [19]. With the processing of NOOP, increases in peak intensity and the formation of new peaks such as 1423 cm^−1^ were observed, which can be attributed to the removal of unwanted components and the addition of new binding functional groups. A similar spectral distribution is also given for CE in Figure 1. The O–H stretching was detected at 3338 cm^−1^, C–H stretching at 2899 cm^−1^, C=C double bond stretching at 1644 cm^−1^, C–O vibrations at 1028 cm^−1^ and 901 cm^−1^, and –COOH interaction at 1429 cm^−1^. After the batch adsorption process, changes such as increases and decreases in peak intensities, as well as the formation of new peaks, were recorded for all samples loaded with ReR-141 (ReR-141+ROP/NOOP/CE). After adsorption, the FTIR spectra of ReR-141+ROP/NOOP/CE samples presented similar functional groups with specific peaks as in unloaded ROP, NOOP, and CE. Some peak fluctuations after adsorption support the retention of ReR-141 molecules on the surfaces of ROP, NOOP, and CE. Notably, peaks at 1442 cm^−1^ and 915 cm^−1^ for ROP; and at 2669 cm^−1^, 1151 cm^−1^, and 952 cm^−1^ for NOOP emerged after adsorption. Additionally, the observation of strong peaks in the 4000–2500 cm^−1^ region may indicate the binding of ReR-141 to the –OH group [24]. The newly appeared and disappeared peaks in the FTIR spectra after adsorption suggest that the removal of ReR-141 primarily occurs through physical adsorption and electrostatic interactions, rather than strong chemical bonding. Specifically, the hydroxyl (–OH) and carboxyl (–COOH) groups present on the surfaces of ROP, NOOP, and CE play key roles in the adsorption mechanism. These groups are capable of forming hydrogen bonds with the sulfonate groups (–SO_3_^−^) of ReR-141. In addition, electrostatic attractions may occur between negatively charged –COO^−^ groups (from deprotonated –COOH) and positively polarized regions of the dye molecule. These interactions are supported by the shifts and intensity changes observed in the FTIR spectra of the adsorbents after adsorption.

### 3.2. Surface Morphology and Element Component of the ROOP, NOOP, and CE

**Figure 2:**
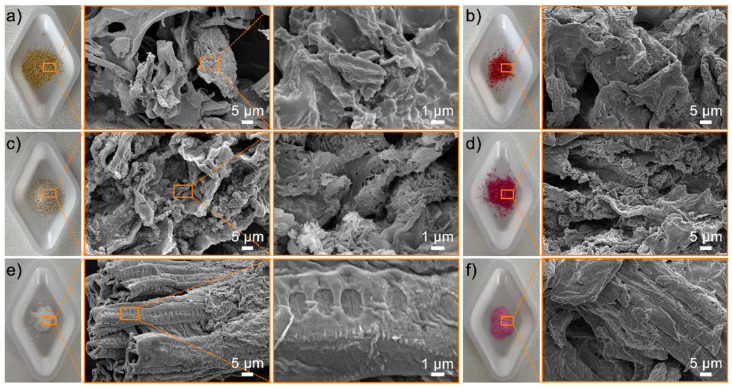
Photographs and SEM images of ROP, NOOP, and CE before and after the adsorption of ReR141: (**a**) ROP, (**b**) ROP-ReR141, (**c**) NOOP, (**d**) NOOP-ReR141, (**e**) CE, and (**f**) CE-ReR141.

The surface morphology of ROP, NOOP, and CE is shown in Figure 2. The compact appearance recorded for ROP is attributed to the drying stage. The surface structure of powdered ROP at different magnifications confirms a heterogeneous structure with pores of various sizes. Pectin is responsible for the fibrous structures found on the surface of ROP [19]. Additionally, NOOP and CE images show a heterogeneous distribution of the surface and a porous formation that facilitates and supports dyestuff adsorption [25,26]. The surface morphology of NOOP recorded a more porous structure, particularly due to the removal of volatile and essential oils found in the flavedo layer. SEM images clearly demonstrate the intensity of pore formation on the surface (Figure 2). Significant morphological changes were observed in the surface morphology of loaded adsorbents obtained after ReR-141 adsorption by ROP, NOOP, and CE. After adsorption, the pores of ROP-ReR141, NOOP-ReR141, and CE-ReR141 samples appeared to be occupied by ReR-141. Particularly for CE, it was determined that pores in areas containing nanofibers were occupied by RER-141.

**Figure 3:**
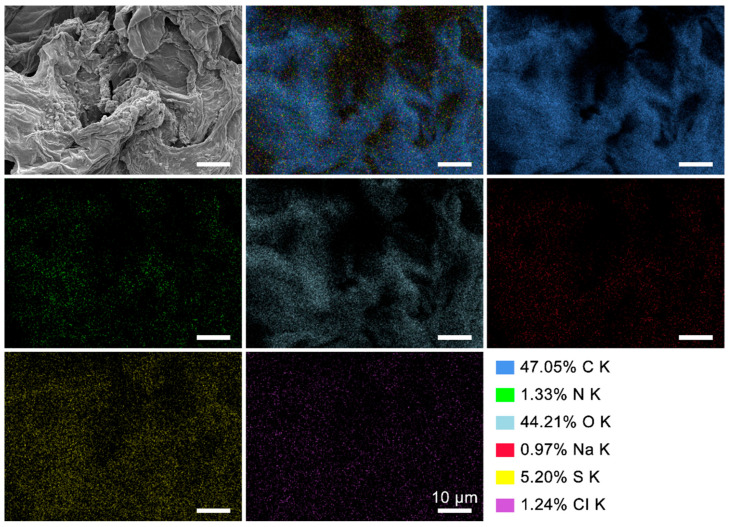
EDX mapping image of NOOP after the adsorption of ReR141.

In addition to SEM analysis for ROP, NOOP, and CE, an EDX analysis was performed to represent the elemental distribution (C, O, N, S, etc.) of the surfaces. In the analysis, the C kα signal at 0.276 keV, the O kα1 signal at 0.523 keV, and the N kα signal at 0.390 keV support the presence of C, O, and N elements [27]. The EDX analysis of both pre- and post-adsorption samples comparatively presents the elemental distribution concerning adsorbent purity and dyestuff binding (Figure S1). The semi-quantitative results in terms of atomic percentage are shown in Table 1. After adsorption, the slight decrease observed in C can be attributed to the partial degradation of lignocellulosic components [28]. The O value, associated with –OH and other oxygen-containing species, was slightly increased after dyestuff adsorption. This can be attributed to the addition of some oxygen-containing functional groups due to the increase in carboxylate distribution. The minimal decrease in N value can be linked to the loss of some amine groups in the medium. Overall, the increase in O and the decrease in N support the formation of new functional groups and the modification of existing ones, which may influence adsorption performance. Mapping was performed for NOOP-ReR141, which exhibited the highest removal efficiency. The mapping images also support the homogeneous binding of the dyestuff to the adsorbent surface (Figure 3).

### 3.3. Effect of pH on Adsorption of ReR-141 Dyestuff

**Figure 4:**
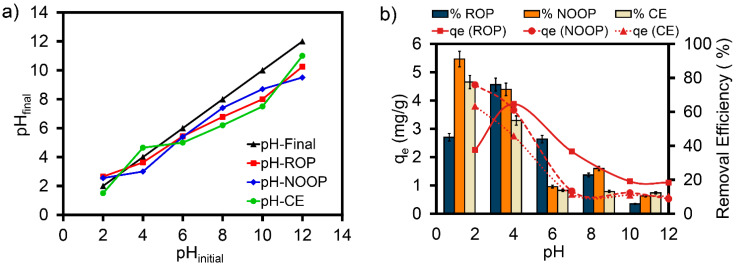
(**a**) pH_zpc_ distribution of ROP, NOOP, and CE; and (**b**) ReR-141 adsorption efficiency of ROP, NOOP, and CE as a function of pH (initial ReR141 concentration, 10 mg/L; ROP, NOOP, and CE concentration, 0.1 g/L; temperature, 20 °C; and contact time, 30 min).

The pH values studied in terms of the surface charge of the adsorbent and the ionization level of the dyestuff, as well as the pH_zpc_ of the adsorbent, are frequently tested in the system. Specifically, changes in the pH factor are also a significant variable for the distribution of hydrogen and hydroxyl ions in the medium. Within the scope of this study, the yield and adsorption capacity (q_e_) of ReR-141 were examined within a pH range of 2 to 12 (Figure 4). Additionally, the functional groups on ROP-NOOP-CE surfaces or ReR-141 are activated according to their protonation and deprotonation capacities by adjusting the pH. This enables retention between ROP-NOOP-CE and ReR-141 molecules through electrostatic interactions. It has been reported that the pH_zpc_ of ROP, NOOP, and CE adsorbents plays a critical role in the process, both in terms of surface charge and the possible mechanism of adsorption [29]. In this study, pH_zpc_ values were determined using the pH drift method. The pH_zpc_ value is a highly important factor, as it influences an adsorbent’s positive and negative charges, the dyestuff’s electrical charge, and its ionization degree [30]. As shown in Figure 4a, the pH_zpc_ values for ROP, NOOP, and CE were determined to be 3.53, 2.66, and 3.07, respectively. Considering the working pH, three different surface charge distributions can be exhibited [31]: (1) At pH < pH_zpc_, ROP, NOOP, and CE surfaces are positively charged, and OH and NH_2_ groups are protonated [32,33]. (2) At pH = pH_zpc_, the surfaces of ROP, NOOP, and CE are neutral (uncharged). (3) At pH > pH_zpc_, ROP, NOOP, and CE surfaces become negatively charged. The obtained pH_zpc_ data are comparable and consistent with the results of adsorption-based removal of different reactive dyestuffs [30,34]. As shown in Figure 4b, the results for ROP indicate that as the pH increases from 4 to 12, the removal efficiency of ReR-141 decreases from 76.03% to 5.82%. Similarly, the q_e_ value decreases from 3.66 mg/g to 0.53 mg/g with increasing pH. At pH values of 7 and above, the binding between the negatively charged ROP surface and ReR-141 molecules weakens. This pH-dependent change is consistent with the pH_zpc_ value of 3.53 for ROP [35,36]. For NOOP, Figure 4b shows that the maximum ReR-141 removal efficiency of 91.10% was achieved at pH 2. Under highly acidic conditions, like pH 2, the NOOP surface is maximally protonated, and since pH (2) < pH_zpc_ (2.66), the NOOP surface charge remains positive, ensuring strong electrostatic interactions with ReR-141 molecules and high adsorption capacity [37,38]. A significant decrease in ReR-141 removal and q_e_ values was observed as pH increased from 2 to 12. Similarly, the maximum removal efficiency of ReR-141 by CE was 77.55% at pH 2. When the pH increased to 4, the ReR-141 removal percentage by CE dropped from 77.55% to 12.3%. Furthermore, as pH increased from 2 to 12, the q_e_ value declined from 3.802 mg/g to 0.615 mg/g. A gradual decrease in dyestuff-removal efficiency was observed with the increasing pH. This decrease can be attributed to the deprotonation (loss of positive charge) of all adsorbent surfaces, leading to reduced electrostatic interactions with ReR-141, a reactive azo dyestuff molecule [38]. Another noteworthy aspect is that, as indicated by FTIR results, acidic carboxyl (–COO) groups (pKa 3.5–5.5) in the structure of ROP, NOOP, and CE are likely the main ligands responsible for binding ReR-141 molecules and may undergo deprotonation [37]. For the removal of dyestuffs like ReR-141 from synthetic water environments, the optimum pH was reported to be between 2 and 6 when different adsorbents were tested [19].

### 3.4. Impact of Adsorbent Dosage

**Figure 5:**
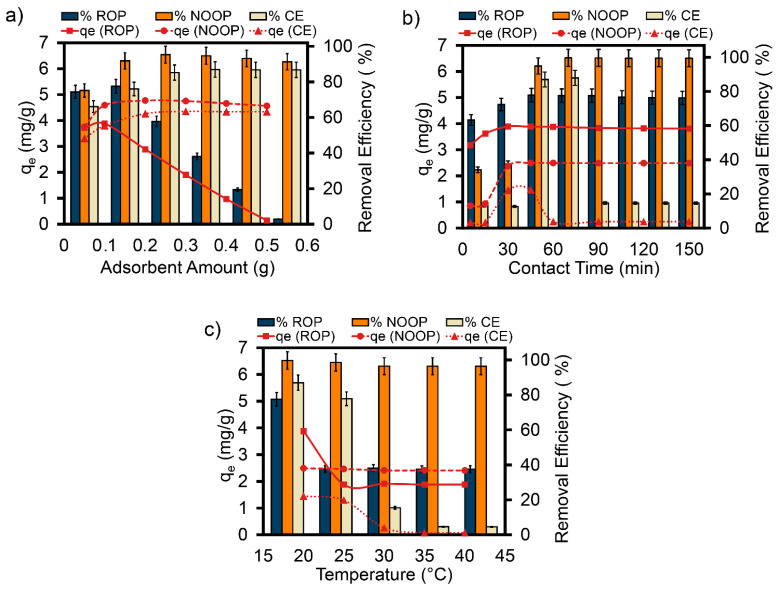
(**a**) ReR-141 removal potential depending on adsorbent dose (initial ReR-141 concentration, 10 mg/L; ROP, NOOP, and CE dose, 0.1 g/L to 0.5 g/L; temperature, 20 °C; and contact time, 30 min). (**b**) ReR-141 removal efficiency depending on contact time (initial ReR-141 concentration, 10 mg/L; ROP dose, 0.1 g/L; NOOP dose, 0.2 g/L; CE dose, 0.3 g/L; temperature, 20 °C; and contact time, 0 min to 150 min). (**c**) ReR-141 adsorption efficiency of ROP, NOOP, and CE as a function of temperature (initial ReR-141 concentration, 10 mg/L; ROP dose, 0.1 g/L; NOOP dose, 0.2 g/L; CE dose, 0.3 g/L; temperature, 20 °C to 40 °C; and contact time, 30 min for ROP, and 45 min for NOOP and CE).

In batch adsorption processes, optimizing the amounts of ROP, NOOP, and CE is essential for the eco-efficient removal of ReR-141. The removal efficiencies and q_e_ values of ReR-141 with ROP, NOOP, and CE were examined at varying dosages of 0.05 to 0.5 g under optimum conditions (10 mg/L ReR-141, 150 rpm, 20 °C) (Figure 5a). The ReR-141 removal percentage decreased with the increasing ROP dosage: from 74.39% (0.05 g/L) to 2.83% (0.5 g/L). This trend may be attributed to saturation of functional groups on the ROP surface, surface area distribution, and the presence of different components (e.g., essential oils) (Figure 5a). At low ROP dosages, ReR-141 molecules disperse more effectively due to a larger available surface area and pore abundance, leading to high q_e_ values. Conversely, at high ROP dosages, interactions between particles reduce total surface area and the slow diffusion of molecules, explaining the decline in q_e_ values. This behavior has also been observed in other adsorption studies [39,40,41]. The maximum adsorption capacities followed the order NOOP > CE > ROP, with values of 4.77, 4.35, and 3.88 mg/g, respectively. As shown in Figure 5a, when NOOP and CE dosages increased from 0.05 g to 0.5 g, ReR-141 removal efficiencies increased from 75.15% to 95.30% (NOOP) and from 66.10% to 86.99% (CE). This increase can be attributed to the higher availability of active surface binding sites with increasing NOOP and CE dosages [42,43,44]. However, beyond 0.3 g, further increases in adsorbent dosage did not significantly enhance ReR-141 elimination effectiveness. The primary goal in adsorption processes is to achieve maximum adsorption capacity (q_m_) with minimal dosage for cost-effectiveness. Therefore, working dosages of 0.1, 0.2, and 0.3 g were selected for ROP, NOOP, and CE, respectively.

### 3.5. Impact of Contact Time

In batch adsorption experiments, contact time between the adsorbent and contaminant molecules is a critical factor in understanding and determining adsorption kinetics [45]. ReR-141 adsorption was investigated for contact times ranging from 5 to 150 min, using ROP, NOOP, and CE adsorbents (Figure 5b). Experimental studies were conducted at 20 °C with 0.1 g ROP, 0.2 g NOOP, and 0.3 g CE dosages at 10 mg/L ReR-141 concentration and pH conditions of pH_ROP_ = 4, pH_NOOP/CE_ = 2. The adsorption process followed two stages: a rapid initial adsorption phase (first 30 min for ROP; 45 min for NOOP and CE), followed by a slower phase approaching equilibrium (see Figure S3). The rapid initial phase is due to abundant available active sites on the adsorbent surfaces, while the subsequent slowdown occurs as these sites become saturated [46].

### 3.6. Thermodynamic Theory Dependent on Temperature

Temperature is a crucial parameter in understanding the thermodynamics of adsorption, the interaction between molecules, and the efficiency of adsorbents. It helps determine whether the system is exothermic or endothermic [47]. In this study, the adsorption of ReR-141 onto ROP, NOOP, and CE was evaluated under optimal conditions at varying temperatures (20, 25, 30, 35, and 40 °C). As illustrated in Figure 5c, the maximum removal efficiencies at 20 °C were recorded as 77.55%, 99.72%, and 86.99% for ROP, NOOP, and CE, respectively. Typically, q_e_ increases with rising temperature due to enhanced reaction rates and the diffusion of dyestuff molecules onto the adsorbent [4]. However, in this study, the q_e_ values reached their peak at 20 °C, followed by a decline as temperature increased. Similar behavior has been observed in other adsorption studies. Silva et al. attributed this decline to the weakening of adsorption forces between dyestuff molecules and the adsorbent surface at elevated temperatures [48]. While the precise mechanism behind the temperature dependence of adsorption is not fully understood, it is thought to result from increased kinetic energy and molecular motion [4]. The observed decrease in q_e_ values is consistent with a physical adsorption process [49].

Thermodynamic parameters (ΔG°, ΔS°, and ΔH°) were calculated using experimental data obtained under different temperature conditions. These parameters were derived from thermodynamic equations presented in Table S2. According to the Van’t Hoff plot (ln K_d_ versus 1/T) shown in Figure S2, the values of ΔH°, ΔS°, and ΔG° were determined from the slope and intercept. Negative ΔH° values indicate exothermic adsorption, while positive values suggest an endothermic process [50]. Table 2 presents the thermodynamic parameters for ReR-141 adsorption. Across the temperature range of 20 °C to 40 °C, all ΔG° values for ROP, NOOP, and CE were negative and showed a decreasing trend, indicating that the adsorption process was spontaneous and thermodynamically favorable. Specifically, the negative ΔG° values suggest strong binding interactions between ReR-141 and the adsorbent surfaces [51]. The ΔG° values observed in this study fell within the range of −20 < ΔG° < 0 kJ/mol, consistent with a physical adsorption mechanism [52]. The process was considered exothermic due to the negative ΔH° values [53]. The fact that the ΔH° values in the adsorption processes involving ROP, NOOP, and CE were below 84 kJ/mol indicates that the process is primarily physical in nature [54]. The positive ΔS° values suggest that ReR-141 was successfully adsorbed onto ROP, NOOP, and CE, transitioning from a more disordered to a more ordered state. This increase in entropy reflects greater randomness and molecular freedom at the solid–liquid interface during the adsorption process [4]. However, based on thermodynamic parameters alone, it is not possible to make definitive conclusions about the reaction mechanism, as the ReR-141 removal process is complex and influenced by multiple functional groups. According to the Arrhenius equation, an activation energy (E_a_) value between 5 and 40 kJ/mol typically indicates physical adsorption, while values between 40 and 80 kJ/mol suggest chemisorption [55]. The calculated Ea values for ROP, NOOP, and CE were 15.969 kJ/mol, 14.674 kJ/mol, and 24.430 kJ/mol, respectively (see Table 2), further supporting the occurrence of physical adsorption.

### 3.7. Adsorption Isotherms and Kinetics

**Figure 6:**
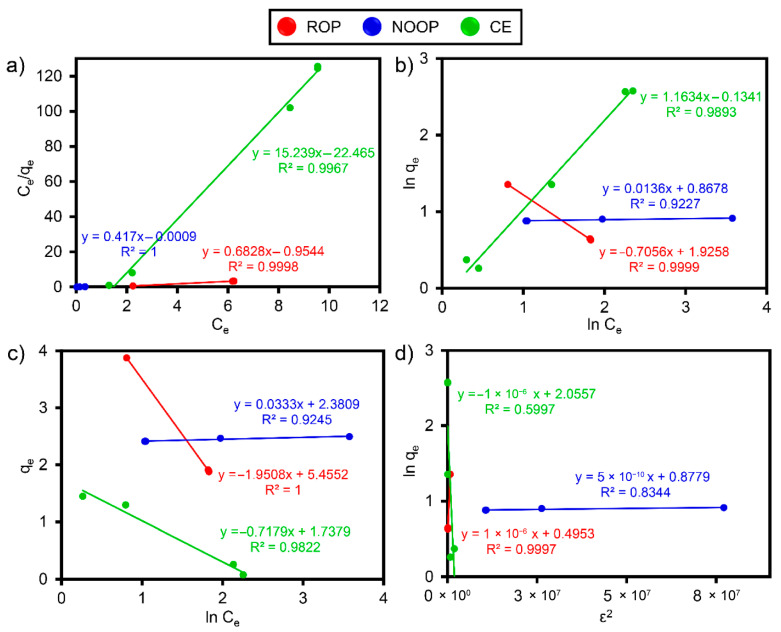
(**a**) Langmuir fitting, (**b**) Freundlich fitting, (**c**) Temkin fitting, and (**d**) D-R fitting.

Different isotherm models describe whether the batch adsorption process is monolayer, multilayer, or energy-based [47]. Isotherms allow the determination of the q_m_ of the selected adsorbents in laboratory-scale experiments [56]. In this study, the isotherm behaviors of ReR-141 on ROP, NOOP, and CE surfaces were evaluated using the equations representing the four isotherms in Table S2. These isotherms are frequently preferred in adsorption processes due to their simplicity and ease of interpretation. The linear graphs are presented in Figure 6, and the corresponding isotherm parameters are provided in Table 3. Regarding the characteristics of the isotherms, Langmuir describes monolayer and homogeneous adsorption on the adsorbent surface [31], while Freundlich reflects the effectiveness of heterogeneous surfaces. The qm values, under the conditions of 150 rpm, 20 °C, and 10 mg/L ReR-141, were calculated as 3.863 mg/g, 4.796 mg/g, and 4.657 mg/g for ROP, NOOP, and CE, respectively. When evaluated according to the equilibrium parameter (R_L_) of the Langmuir isotherm (using the equation in Table S2), R_L_ values ranged between 0.02 and 0.128 for ROP, NOOP, and CE. These R_L_ values indicate that the adsorption process of ReR-141 by ROP, NOOP, and CE is favorable (0 < R_L_ < 1). Based on the R^2^, the Freundlich is considered the most suitable isotherm after the Langmuir. The Freundlich isotherm indicates greater heterogeneity compared to Langmuir [56]. The 1/n (1/n = 0 indicates that the process is irreversible, 0 < 1/n < 1 indicates a favorable process, and 1/n > 1 suggests an unfavorable process) Freundlich factor is essential for determining the type of adsorption [57]. In this study, under ideal conditions, the 1/n values for ROP, NOOP, and CE were 0.706, 0.274, and 0.980, respectively, all within the range of 0 < 1/n < 1, indicating efficient adsorption and rapid removal of ReR-141.

The Temkin isotherm provides an energy-based modeling approach. According to the Temkin isotherm, the B value (kcal/mol) can be used to identify the adsorption type [58]. When 1 < B < 20, physical and chemical adsorption may occur simultaneously. The B values for ROP, NOOP, and CE were calculated as 12.486, 7.316, and 3.393 kcal/mol, respectively, confirming the coexistence of physi- and chemisorption. Based on the R^2^ values, the order of the best-fitting isotherms for the batch experiment data is as follows: Langmuir > Freundlich > Temkin > DR. The similar R^2^ values for the adsorption of ReR-141 molecules on ROP, NOOP, and CE surfaces suggest that the adsorption process initially involves monolayer adsorption on active surface sites, followed by multilayer adsorption. Furthermore, it was observed that the tested isotherms overlap graphically (Figure 6), which can be attributed to the fact that many isotherms are variations of the Langmuir and Freundlich. Many studies in the literature report on the importance of selecting appropriate isotherms for interpreting experimental data from batch adsorption processes [30,59,60].

**Figure 7:**
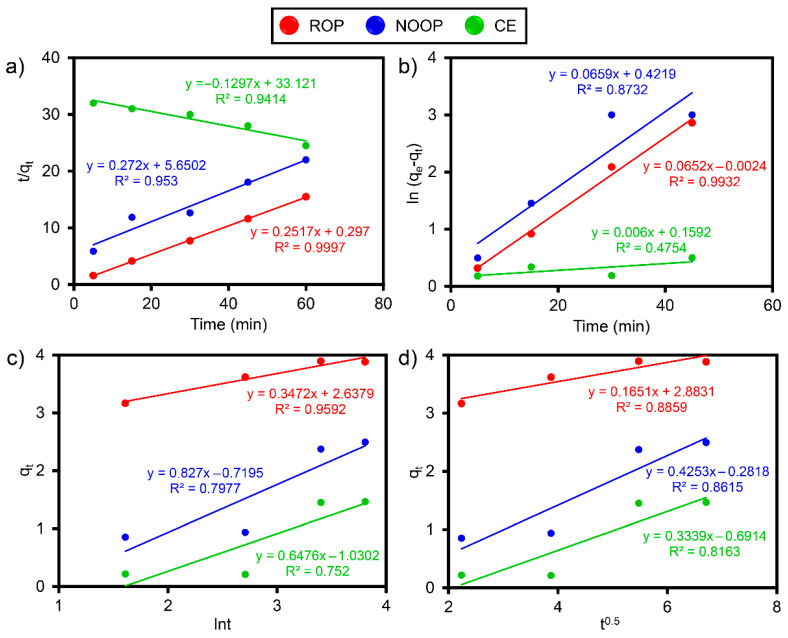
(**a**) PFO fitting, (**b**) PSO fitting, (**c**) Elovich fitting, and (**d**) IPD fitting.

The selection of a kinetic model provides critical information regarding the rate and structure of adsorption [61]. Four kinetics were applied in this investigation to comprehend the behavior and the rate: PFO, PSO, Elovich, and IPD (see Table S2). For each kinetic model, the kinetic constants representing the linear relationship were determined from the intersection points and slopes of the graphs (Figure 7). The graphical representations of the models and the kinetic parameters derived from the equations are presented in Figure 7 and Table 3. Under the conditions of 10 mg/L ≤ ReR-141 ≥ 10 mg/L, the PFO and PSO models generally provided better fits to the adsorption process [62]. The results indicate that the Elovich and IPD are insufficient to explain the ReR-141 adsorption. Although the Elovich model is typically compatible with heterogeneous adsorbent surfaces, the greater homogeneity of the ROP, NOOP, and CE surfaces in this study suggests that the model is unsuitable. The R^2^ values indicate that the PSO model provides the best fit compared to the other kinetic models [63]. The R^2^ values indicate that the PSO kinetic model provides a better fit compared to other kinetic models. This finding aligns with the rapid adsorption behavior observed. During the adsorption process, physisorption typically occurs via weak van der Waals forces on the adsorbent surface, while chemisorption involves stronger interactions through electron exchange. The PSO model accounts for both potential adsorption mechanisms [64]. Based on the PSO data, it can be inferred that adsorption may involve electron exchange between the adsorbent surface and the pollutant. However, since the PSO model does not distinguish between chemical and physical adsorption under equilibrium conditions, the presence of multiple mechanisms cannot be conclusively determined based on kinetic results alone.

### 3.8. Evaluation in Relation to Other Adsorbents

Table 4 provides a summary of how well ROP, NOOP, and CE removed the dye ReR-141 compared to other adsorbents used for similar purposes. The findings indicate that ROP, NOOP, and CE exhibit promising potential for the treatment of both synthetic and real-scale wastewater containing ReR-141. Specifically, ROP and its derived adsorbents demonstrate favorable characteristics, such as high availability in the receiving environment, low cost, and effective elimination of target dyestuffs from water-based media, suggesting their potential as alternative adsorbents.

### 3.9. Potential Mechanism of Adsorption

**Figure 8:**
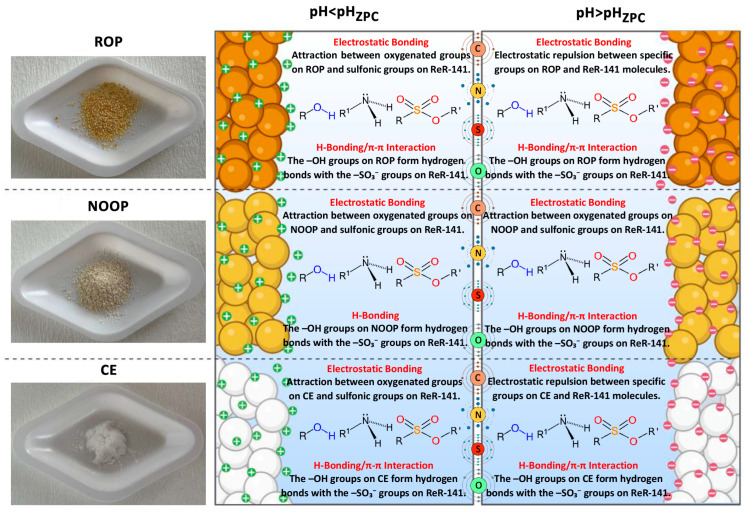
Possible adsorption mechanism of ReR-141 onto ROP, NOOP, and CE.

The possible adsorption mechanism for any adsorption process may occur through the combination of one or more mechanisms. These mechanisms generally include electrostatic interactions, hydrogen bonding, complex formation, and ion exchange between the adsorbent and the adsorbate [8,69]. Such mechanisms are influenced by various factors during the process. In particular, the functional groups inherent to ROP, NOOP, and CE play a significant role in guiding the adsorption mechanism. Hence, the mechanism can be predicted based on FTIR analysis. Orange peel, the base material for ROP, is rich in cellulose, lignin, and hemicellulose, which contain abundant -OH and -OCH_3_ functional groups. These functional groups are key contributors to the adsorption mechanism and are crucial in the removal of dyestuff molecules [31]. Furthermore, kinetic models and thermodynamic results can also aid in predicting the underlying adsorption mechanism. Another crucial factor affecting the mechanism is the solution pH and pH_zpc_ of the adsorbents. As discussed in the “Effect of pH” section, changes in electrostatic interactions are associated with pH and pH_zpc_ values. As shown in Figure 8, reactive dyestuffs such as ReR-141 contain sulfonate (R-SO_3_^−^) groups, and the following reactions occur in an aqueous environment based on these groups [11,70]:

(4) $$ReR-141SO_{3}Na\to ReR-141-SO_{3}^{-}+{Na}^{+}$$

(5) $$\frac{\frac{ROP}{NOOP}}{CE}+H^{+}\to \frac{\frac{ROP}{NOOP}}{CE}---H^{+}$$

(6) $$\frac{\frac{ROP}{NOOP}}{CE}+H^{+}+ReR-141-SO_{3}^{-}\to \frac{\frac{ROP}{NOOP}}{CE}--SO_{3}^{-}-ReR-141+H^{+}$$

When pH < pH_zpc_, strong electrostatic attraction is expected between the positively charged surfaces of ROP, NOOP, and CE and the -SO_3_^−^ groups in the ReR-141 structure (Figure 8). However, when pH > pH_zpc_, electrostatic repulsion may occur due to the negatively charged surfaces of ROP, NOOP, and CE and the ReR-141 molecules, leading to a decrease in the dyestuff-removal efficiency. Beyond electrostatic interactions, other mechanisms may also be involved in the current adsorption process. According to FTIR data, the -NH and -OH groups on the surfaces of ROP, NOOP, and CE may act as hydrogen donors, while the -SO_3_ and amine groups in the ReR-141 molecules may serve as hydrogen acceptors, facilitating dipole–dipole hydrogen bonding (H-bonding). Additionally, n-π interactions between the oxygen atoms on the adsorbents and the π-system in the ReR-141 ions represent another potential adsorption mechanism [1]. Moreover, π-π interactions may occur between the electron-donating carbon on ROP, NOOP, and CE and the electron-accepting amine groups of ReR-141. Lastly, another plausible process is the transfer of ions within the ReR-141 molecules and the O, N, and C atoms on the adsorbents. Therefore, the possible adsorption mechanism of ReR-141 onto ROP, NOOP, and CE adsorbents is controlled by electrostatic interactions, H-bonding, n-π interactions, π-π interactions, and ion exchange.

### 3.10. Desorption Capacity of ROP, NOOP, and CE

**Figure 9:**
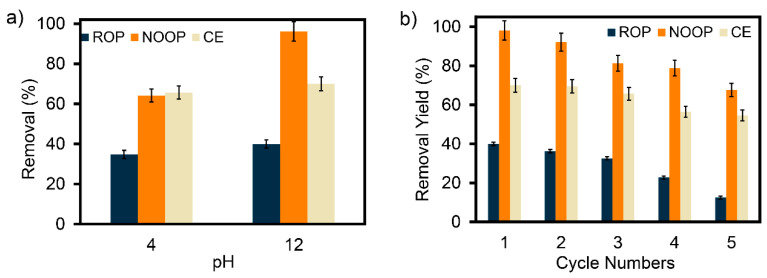
(**a**) Selection of eluents for the desorption of ROP, NOOP, and CE. (**b**) Desorption efficiency of saturated ROP, NOOP, and CE after five consecutive desorption cycles.

In the adsorption process, desorption is essential to maintain economic feasibility and to prevent secondary pollution caused by contaminated adsorbents. In this study, 0.2 M NaOH and HCl solutions were used as eluents to determine the optimal desorption conditions. For the removal of ReR-141 by ROP, NOOP, and CE, the laboratory-scale desorption procedure is illustrated in Figure S3. A preliminary experiment was conducted under pH 12 (NaOH) and pH 4 (HCl) conditions to evaluate the desorption capacities of ROP, NOOP, and CE (Figure 9a). Since the surface characteristics of ROP, NOOP, and CE tend toward acidity, NaOH was found to be the most effective desorption agent for all adsorbents. Therefore, subsequent experiments were carried out using 0.2 M NaOH (Figure 9b). Among the adsorbents, NaOH had the least impact on ROP, with a maximum desorption efficiency of 39.97% in the first cycle. The highest desorption efficiency in the first cycle was observed for NOOP at 98.16%, while CE exhibited a maximum desorption efficiency of 70%. As shown in Figure 9b, even after five consecutive desorption cycles with ReR-141, ROP, NOOP, and CE demonstrated remarkable regenerative performance. A decrease in desorption efficiency was observed for all adsorbents after the third cycle. Compared to the first cycle, decreases of 18.54%, 17.25%, and 6.14% were observed for ROP, NOOP, and CE, respectively. This slight decline is primarily attributed to the residual ReR-141 that was previously adsorbed and not fully desorbed, leading to accumulation within the adsorbents. Although promising results were achieved in the first two cycles, further optimization and reuse studies of ROP, NOOP, and CE using different eluents are recommended. In conclusion, ROP, NOOP, and CE exhibit relatively stable desorption performance over multiple cycles for ReR-141.

## 4. Conclusions

This study demonstrates the potential of ROP, NOOP, and CE as functional and wieldy adsorbents for the yield of ReR-141 dyestuff. NOOP exhibited the highest removal efficiency (99.72%) due to its increased surface area and structural categories, while ROP and CE also showed significant adsorption capacities. The PSO kinetic model provided a good description of the adsorption system and reported the Langmuir equation the best, indicating monolayer coverage. Thermodynamic studies verified that the reaction was exothermic and random. Desorption data indicated that NOOP retained 98.16% yield after trio series, demonstrating excellent reusability. The main surface contact mechanisms involve hydrogen bonding, π–π interactions, and electrostatic forces. This work suggests that orange peel derivatives, particularly NOOP, are promising, low-cost, and environmentally friendly materials for dyestuff-removal applications, contributing to sustainable waste management and water treatment practices.

## Figures and Tables

**Table 1:** EDX analysis results of ROP, NOOP, and CE before and after the adsorption of ReR141.

Samples	C K (%)	N K (%)	O K (%)	Na K (%)	S K (%)	CI K (%)
ROP	52.64	4.91	42.45	-	-	-
ROP-R141	50.22	2.57	43.12	0.31	2.94	0.84
NOOP	53.53	3.23	43.24	-	-	-
NOOP-R141	47.05	1.33	44.21	0.97	5.20	1.24
CE	50.70	3.99	45.31	-	-	-
CE-R141	47.54	2.05	46.06	0.40	3.14	0.81

**Table 2:** ∆H°, ∆G°, and ∆S° results for ROP, NOOP, and CE.

Parameters	Unit	ROP	NOOP	CE
ΔH°	(kJ/mol)	−24.28	−30.15	−25.29
ΔS°	(J/mol/K)	9.08	7.91	8.63
ΔG°_293.15_	(kJ/mol)	−5.95	−14.07	−18.11
ΔG°_298.15_	(kJ/mol)	−5.84	−10.97	−14.97
ΔG°_303.15_	(kJ/mol)	−5.72	−9.40	−13.93
ΔG°_308.15_	(kJ/mol)	−5.64	−9.35	−13.30
ΔG°_318.15_	(kJ/mol)	−4.87	−9.32	−12.26
E_a_	(kJ/mol)	15.97	14.67	24.43

**Table 3:** Kinetic and isotherm parameters for ROP, NOOP, and CE, presented with 95% confidence intervals.

**Optimum Kinetics Results for ReR-141**				
	**Parameter**	**ROP**	**NOOP**	**CE**
PSO	k_2_ (g/mg/min)	2.13 × 10^−1^ ± 0.044	1.31 × 10^−1^ ± 0.072	5.08 × 10^−5^ ± 0.147
	q_e_ (mg/g)	3.973 ± 0.043	3.976 ± 0.051	3.312 ± 0.0360
	R^2^	0.999	0.953	0.941
PFO	k_1_ (1/min)	0.065 ± 0.013	0.066 ± 0.016	0.006 ± 0.001
	R^2^	0.993	0.873	0.475
Elovich	α	1.99 × 10^3^ ± 1.0045 × 10^4^	2.39 × 10^0^± 2.15 × 10^6^	4.91 × 10^0^ ± 2.32 × 10^10^
	β	0.3472 ± 0.175	0.8270 ± 0.416	0.6476 ± 0.341
	R^2^	0.752	0.797	0.959
IPD	k_ipd_	0.1651 ± 0.075	0.4253 ± 0.192	0.3339 ± 0.153
	R^2^	0.816	0.861	0.886
**Optimum Isotherm Results for ReR-141**				
	**Parameter**	**ROP**	**NOOP**	**CE**
Langmuir	q_m_, mg/g	3.863 ± 1.032	4.796 ± 1.304	4.657 ± 1.266
	K_L_, 1/mg	0.715 ± 0.399	4.633 ± 2.586	0.678 ± 0.378
	R_L_	0.123 ± 0.033	0.021 ± 0.005	0.128 ± 0.047
	R^2^	0.999	1.000	0.997
Freundlich	K_F_, ((mg/g)(L/mg)^1/n^)	6.861 ± 3.816	2.382 ± 1.614	1.144 ± 0.775
	n	1.417 ± 0.129	3.646 ± 0.210	1.020 ± 0.093
	1/n	0.706 ± 0.064	0.274 ± 0.016	0.980 ± 0.089
	R^2^	0.999	0.923	0.989
D-R	K_DR_, (mol k)^−1^)^2^	1.00 × 10^−4^ ± 0.022	5.00 × 10^−4^ ± 0.058	1.00 × 10^−3^ ± 0.026
	R^2^	0.997	0.834	0.599
Temkin	K_T_, L/mg	16.39 ± 4.097	13.91 ± 3.477	1.27 ± 0.711
	B (kcal/mol)	12.486 ± 2.687	7.316 ± 1.574	3.393 ± 0.736
	R^2^	1.000	0.925	0.982

**Table 4:** Comparison of % removals for ROP, NOOP, and CE with some reported different adsorbents.

Adsorbent	pH	Adsorbent (g)	Time (min)	Temperature (°C)	% Removal	References
Cotton fibers	3.00	0.25	70	20	96.87	[11]
Pecan nutshell	3.00	0.05	-	25	85.00	[65]
Ash Waste	6.00	0.50	60	25	97.00	[66]
Leaf sheath waste	2.00	1.00	180	-	72.70	[67]
γ-Al_2_O_3_ nanopart.	4.81	0.38	51.61	-	97.74	[35]
Cuttlebone	6.50	1.20	120	24	90.00	[68]
ROP	4.00	0.10	30	20	77.85	This study
NOOP	2.00	0.20	45	20	99.72	
CE	2.00	0.30	45	20	87.92	

## Data Availability

All data are available in the manuscript or electronic Supplementary Materials.

## Supplementary Materials

The following supporting information can be downloaded at: https://www.mdpi.com/article/10.3390/polym17131875/s1. References [71,72,73,74,75,76,77,78,79] are cited in the Supplementary Materials.
