# Supplementary material for: Strong Amphoteric Adsorption of Reactive Red-141 onto Modified Orange Peel Derivatives: Optimization, Characterization, and Mechanism
Source: Polymers (Basel). 2025 Jul 4;17(13):1875. doi: 10.3390/polym17131875 (PMC12252381; doi:10.3390/polym17131875)
Supplement: Supplementary file 1 [file polymers-17-01875-s001.zip › polymers-3707141-supplementary.pdf]

Supplementary Materials

Strong Amphoteric Adsorption of Reactive Red-141 onto Modified Orange Peel Derivatives: Optimization, Characterization, and Mechanism

Table S1. Specific properties of ReR-141 dyestuff.

Properties	ReR-141 dyestuff
Molecular formula	$C_{52}H_{26}Cl_2N_{14}Na_8O_{26}S_8$
Molecular weight	1774.1852 (g/mol)
Chemical structure	

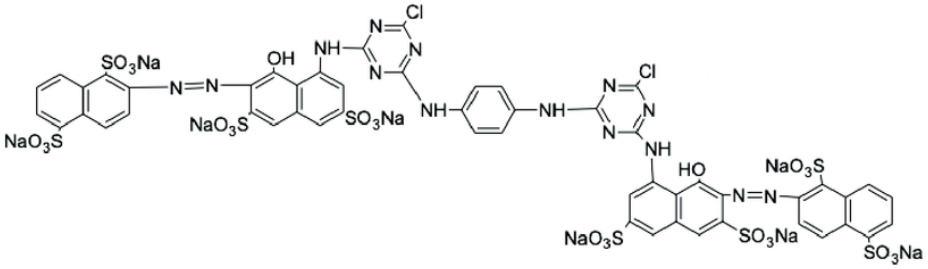

Table S2. Equations used in ReR-141 adsorption on ROP, NOOP, and CE [1-9].

Kinetic equations		
Model	Equation	Parameters
PFO	$\log(q_{eq,1} - q_t) = log q_e - \frac{k_1}{2.303} t$	$q_{eq,1}$ (mg/g): biosorption capacity at equilibrium<br>k_1 (min^{-1}): apparent rate constant of PFO
PSO	$\frac{t}{q_t} = \frac{1}{q_{eq,2}^2 k_2} + \frac{1}{q_e} t$	$q_{eq,2}$ (mg/g): biosorption capacity at equilibrium<br>k_2 (g/mg.min): apparent rate constant of PSO
IPD	$q_{(t)} = k_{int.}\ t^2 + C$	k_{int}: intra-particle diffusion rate constant (in mg/g.$min^{1/2}$).
Elovich	$q_t = \frac{1}{\beta} \ln(1 + \alpha\beta t)$	α: initial adsorption rate (mg/g/min) and β: surface coverage constant (g/mg)
**Isotherms equations**		
Model	Equation	Parameters
Langmuir	$\frac{C_e}{q_e} = \frac{C_e}{Q_{max}} + \frac{1}{K_L\ Q_{max}}$<br>$R_L = \frac{1}{1 + K_L C_0}$	q_m, (mg/g): biosorption capacity at saturation of monolayer<br>K_L (L/mg): affinity coefficient<br>R_L: equilibrium parameter
Freundlich	$\log\ q_e = \log K_f - \frac{1}{n} \log C_e$	K_F and n: empirical parameters of Freundlich equation

Temkin	$q_{eq} = \frac{RT}{b_T}\ln C_e - \frac{RT}{b_T}\ln A_T$	A_T (L/mg): equilibrium binding capacity<br>b_T (in J/kg.g^2): Temkin constant addressing the sorption heat<br>R (is the gas constant=8.314 J.mol/K)
D-R	$\ln q_e = \ln q_m - K_{DR}\,(\epsilon^2)$	K_{DR}: specific D-R constant; Ɛ: Polanyi potential
**Thermodynamic Equations**		
Model	Equation	Parameters
Gibbs energy/ enthalpy/entropy	$\Delta G^o = -RT ln K_d$<br>$\Delta G^o = \Delta H^o - T\Delta S^o$	R: universal gas constant (8.314 J/mol/K); T: solution temperature (K), kd: adsorption affinity. ΔH: enthalpy, ΔS: entropy. ΔG: standard free energy, E_a: activation energy.
Van´t Hoff equation	$lnK_d = \frac{\Delta S^0}{R} - \frac{\Delta H^0}{RT}$<br>$K_d = \frac{Q_e}{C_e}$	
Arrhenius equation	$\ln k = \ln A - \frac{E_a}{RT}$	

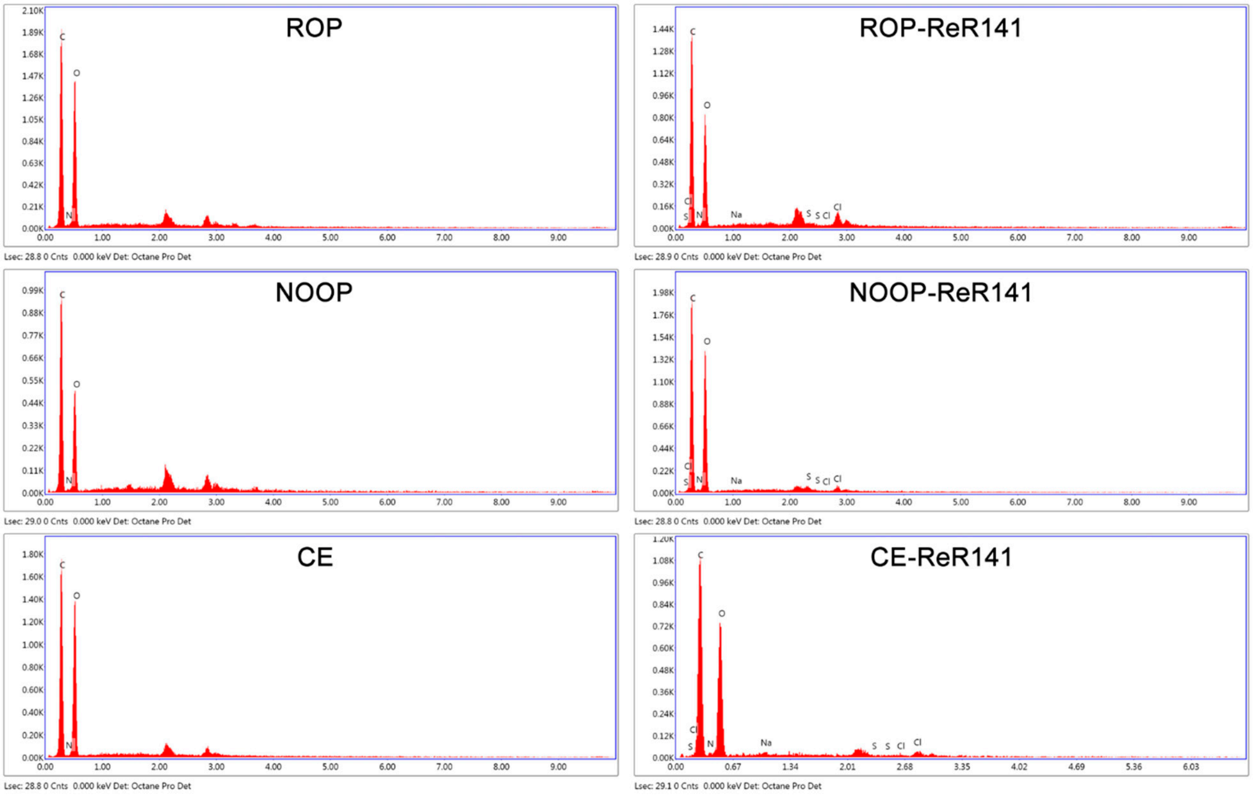

Figure S1. EDX analysis results of ROP, NOOP, and CE before and after adsorption for ReR141.

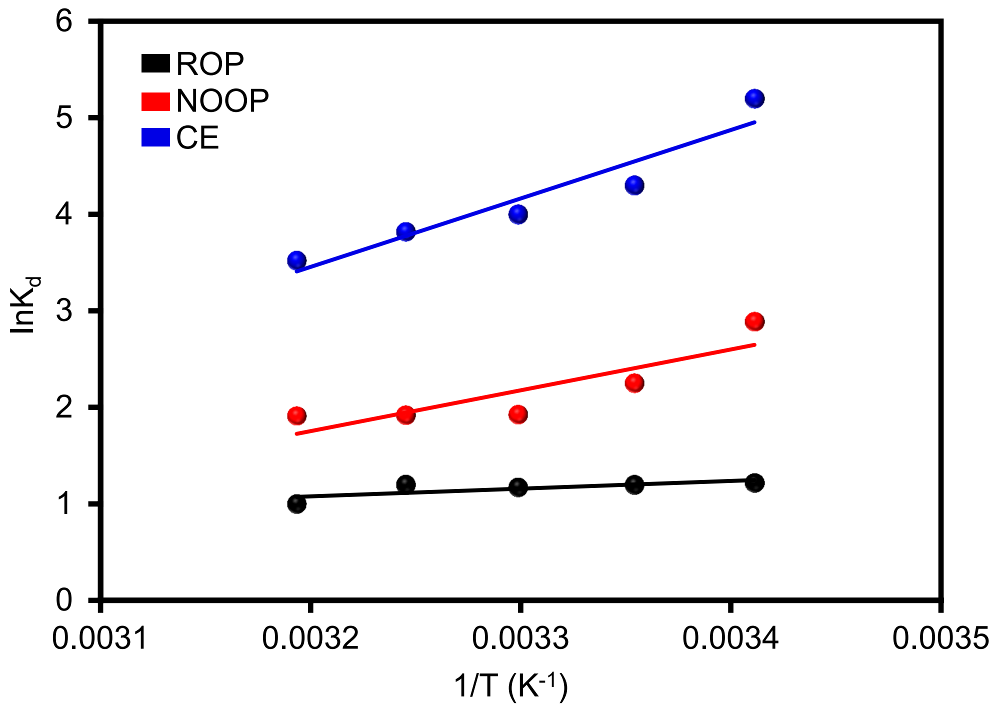

Figure S2. The impact of temperature on the removal of ReR141 by ROP, NOOP, and CE: thermodynamic analysis.

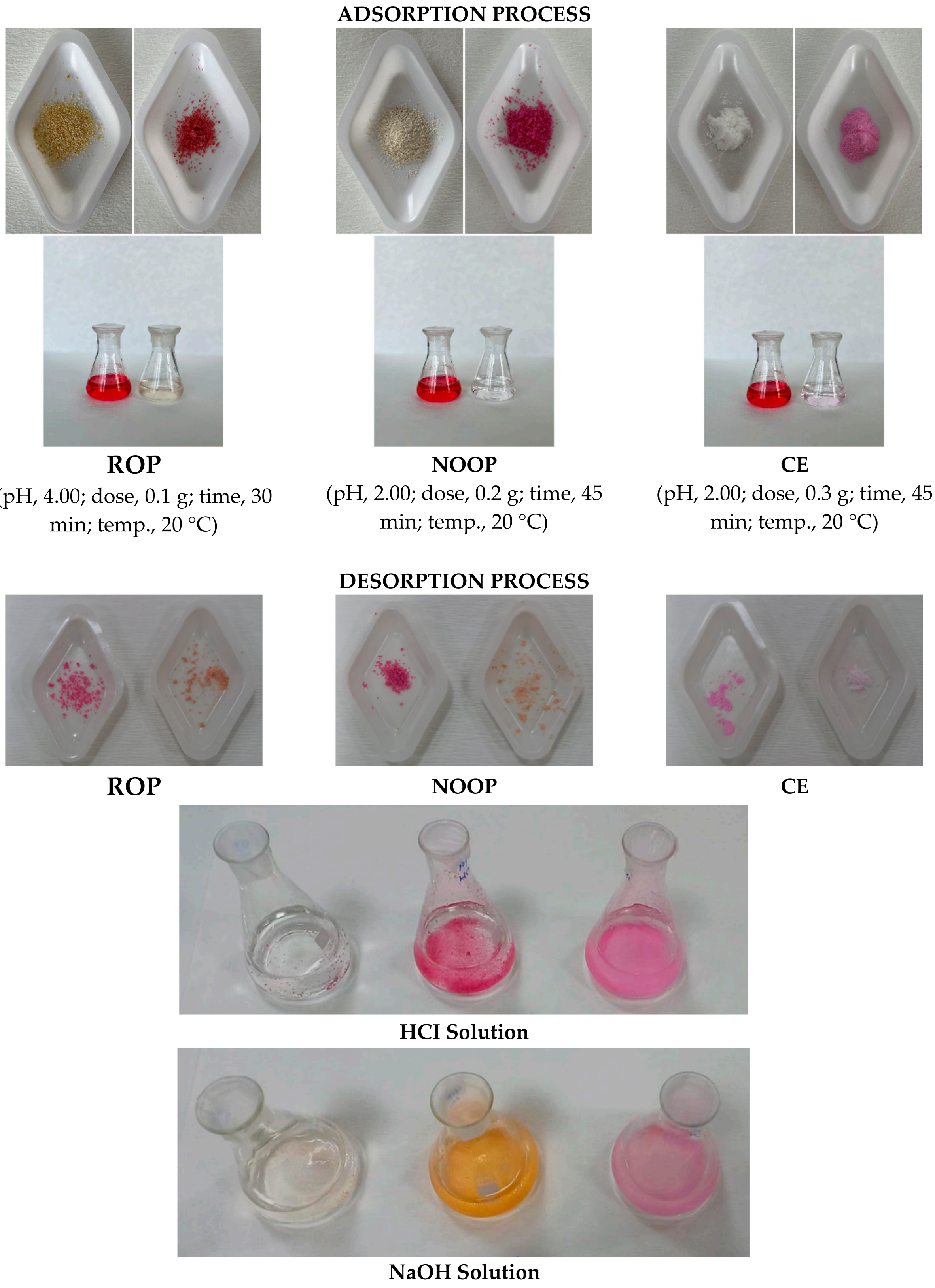

Figure S3. Adsorption and desorption processes on the removal of ReR141 by ROP, NOOP, and CE under optimum conditions.

References

[1] M. Dubinin, The equation of the characteristic curve of activated charcoal, Dokl. Akad. Nauk. SSSR., 1947, pp. 327-329.
[2] S.Y. Elovich, O. Larinov, Theory of adsorption from solutions of non electrolytes on solid (I) equation adsorption from solutions and the analysis of its simplest form,(II) verification of the equation of adsorption isotherm from solutions, Izv. Akad. Nauk. SSSR, Otd. Khim. Nauk 2(2) (1962) 209-216.
[3] H. Freundlich, Über die adsorption in lösungen, Zeitschrift für physikalische Chemie 57(1) (1907) 385-470.
[4] K.R. Hall, L.C. Eagleton, A. Acrivos, T. Vermeulen, Pore-and solid-diffusion kinetics in fixed-bed adsorption under constant-pattern conditions, Industrial & engineering chemistry fundamentals 5(2) (1966) 212-223.
[5] Y.-S. Ho, G. McKay, Pseudo-second order model for sorption processes, Process biochemistry 34(5) (1999) 451-465.
[6] S. Lagergren, Zur theorie der sogenannten adsorption geloster stoffe, Kungliga svenska vetenskapsakademiens. Handlingar 24 (1898) 1-39.
[7] I. Langmuir, The constitution and fundamental properties of solids and liquids. Part I. Solids, Journal of the American chemical society 38(11) (1916) 2221-2295.
[8] M.J. Temkin, V. Pyzhev, Recent modifications to Langmuir isotherms, (1940).
[9] W.J. Weber Jr, J.C. Morris, Kinetics of adsorption on carbon from solution, Journal of the sanitary engineering division 89(2) (1963) 31-59.
